# Broken-Edge Decision-Making Strategy for COVID-19 over Air Railway Composite Network

**DOI:** 10.1155/2022/4149477

**Published:** 2022-01-18

**Authors:** Hui Sun, Yicong Qin, Zhicheng Mu, Rui Wang

**Affiliations:** College of Electronic Information and Automation, Civil Aviation University of China, Tianjin 300300, China

## Abstract

In order to control the spread of the COVID-19 virus, this study proposes an ARCN-SUTS (air railway composite network susceptible-untested-tested-susceptible) model based on the correlation characteristics of the air railway composite network in mainland China. Furthermore, this study also puts forward a broken-edge decision-making strategy for the purpose of making decision about the edge efficiently broken and avoiding the second outbreak of the virus spread to minimize the economic losses for railway and civil aviation companies. Finally, simulation results demonstrate that the proposed strategy can effectively control the spread of the virus with minimal economic losses.

## 1. Introduction

Since December 2019, a typical human-to-human epidemic of novel coronavirus has broken out. At the beginning of the outbreak, the Chinese government attached great importance to this event. The Chinese government not only rapidly deployed various medical and rescue departments but also adopted effective joint prevention and control measures. Wuhan was locked down. Not only that, all cities, counties, and districts in China adopted prohibition measures to limit the flow of population to the greatest extent in order to achieve the purpose of curbing the spread of the epidemic. Till 24:00 on June 30, 2020, the total number of confirmed cases in the world was 10,185,374, and the cumulative death toll was 503,862 including 83,534 confirmed cases and 4,634 deaths in China [1, 2]. The average daily number of new cases in China at present has leveled off; however, the possibility of a rebound has not been ruled out [3–5]. From reference [6], it is known that the total passenger turnover in 2020 is 1,925,147 million person kilometers (pkm), where passenger turnover of Chinese civil aviation and railway is 145,747 million pkm in total accounting for 75.7% of the total passenger turnover. In order to guarantee the recovery of the social and economic order in China, it is necessary to restrict the traffic flow in civil aviation and railway transportation and find a feasible decision-making strategy to control the epidemic spread within a reasonable threshold through the combination transportation network among aviation and high-speed railway (HSR). It is necessary to make full use of the Chinese civil aviation and HSR transportation system and realize the maximization of transportation efficiency and economic benefits.

Studies on the spread of the virus in a complex network model have been going on for decades; references [7, 8] not only introduced classic virus propagation models and classifications but also made comparisons about their advantages and disadvantages. In particular, the worldwide spread of COVID-19 has inspired scholars to study the spreading models of the COVID-19 virus. References [9–11] introduced that the infected persons show some latent characteristics in the initial stage according to the characteristics of COVID-19 virus propagation. The explored nodes are added into the basic susceptible-infected-recovered (SIR) framework, and the immune loss area and death zone are inserted into the basic SIR framework to establish the susceptible-explored-infected-recovered-susceptible (SEIRS) model. References [12, 13] introduced that the threshold property of the basic reproduction number was introduced for the propagation model of age-dependent SIS (susceptible-infection-susceptible) diseases in heterogeneous networks. The introduction of a popular threshold can accurately grasp the control effect of the network. Reference [14] proved that no critical immune threshold was caused by the infinite connectivity fluctuation of scale-free networks. References [15, 16] analyzed the controllability of complex networks by combining graph theory and using the controllability theory of linear systems. Emergency decision-making on COVID-19 has become one of the hot issues in recent years. The aim of emergency decision-making is to reduce casualties and property losses[17,18]. Emergency decision-making could be viewed as a multiattribute decision-making problem due to many aspects taken into consideration [19–21]. Reference [22] investigated a biobjective fuzzy emergency distribution center location model for reflecting the urgency and uncertainty of large-scale emergencies. Reference [23] developed a method for interactive multicriteria group decision-making with probabilistic linguistic term sets and applied it to the emergency assistance area selection of COVID-19 for Wuhan. Furthermore, it is also necessary to strengthen research on dynamic emergency decision-making for the COVID-19 spread based on traffic networks. It is known that a traffic network is a kind of complex network. The spread of the virus in the field of transportation is different from other biological viruses, computer viruses, and information dissemination models over social networks. The mobility of personnel in the field of the transportation network is strong, and the network is highly complex. From a global perspective, economic development has increasingly demanded air-rail combined transport, which has also led to a more complex transportation network. The associated research studies have been globally carried out. From the cooperation perspective, reference [24] proposed a model of aviation and railway intermodal transportation, which can obtain better-integrated transportation services and economic benefits with lower environmental costs. In response to the current situation of China's aviation and railway [25–27] analyzed the complex characteristics of the air railway composite network and constructed a mixed-integer programming model for the air railway composite network with the lowest intermodal cost. It is well known to all that the delay propagation of HSR train or flight over the network can be controlled by using complex network theory and propagation dynamics model during emergencies [28–30]. Similarly, the combination of complex network theory and propagation model is applicable to control the spread of biological viruses over the transportation network. References [31, 32] improved the SEIR (susceptible-explored-infected-recovered) and SEI (susceptible-explored-infected) models based on the spreading characteristics of COVID-19 with system dynamics in epidemic areas. However, this study does not propose the strategy of network edge breaking so as to reduce the virus transmission rate and economic loss. Therefore, this study establishes the COVID-19 virus propagation dynamics model over the air railway composite network and studies the related broken-edge decision-making strategies to control the virus propagation among cities. Simultaneously, this study considers the epidemic threshold of the composite heterogeneous network of air and railway to ensure that the virus propagation among cities always lies within the epidemic threshold range over the network. This can control virus propagation over the air railway composite network. This study also studies the corresponding optimal strategy for the economic consideration of the aviation and railway corporations so as to minimize economic losses within the controllable virus propagation threshold range.

There are two main contributions in this study that are summarized as follows:

1. First, based on the susceptible-exposed-infected-susceptible (SEIS) model [33], the air railway composite network susceptible-untested-tested-susceptible (ARCN-SUTS) model is designed to describe the spread dynamics of the COVID-19 virus among city nodes.
2. Second, in order to avoid the second outbreak of the epidemic and minimize the economic loss over the network, the broken-edge decision-making strategy is proposed to control the spread of the virus in the composite network with less time and minimum economic losses.

The rest of this study is organized as follows. The construction and characteristic analyses of the composite network are introduced in Section 2. The ARCN-SUTS propagation dynamics model is developed and presented in Section 3 in detail.  Section 4 presents a broken-edge decision-making strategy over the air railway composite network.  Section 5 conducts simulations that demonstrate the effectiveness of the broken-edge decision-making strategy. Conclusions are drawn in the Section 6.

## 2. The Construction and Characteristic Analysis of the Composite Network

With the expansion of the scale of the air railway composite network, it owns higher complexity and lower controllability; therefore, the study of network complexity is particularly important. This section analyzes the characteristics of mainland China HSR subnetwork, aviation subnetwork, and air railway composite network. Data come from the websites [34, 35]. This study considers all nationwide cities at the prefecture level and above including 223 cities with HSR transport capacity and 155 cities with aviation transport capacity. Any city with HSR or civil aviation transport capacity is considered as a city node on the air railway composite network, and 261 city nodes in total over the composite network are obtained. In this composite network, if there is an HSR line between any two neighboring cities or a direct flight between any two cities, a connection between these two cities is created.

**Figure 1:**
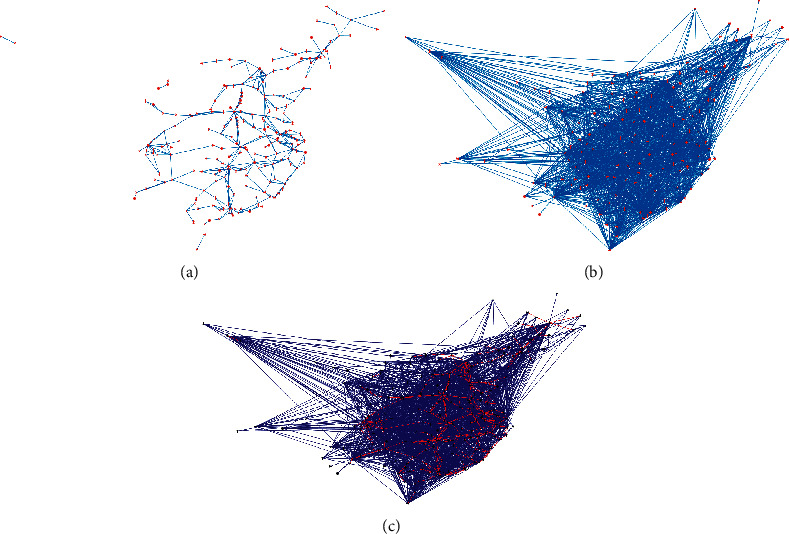
Subnetwork topologies and composite network topology for air railway systems in mainland China. (a) China HSR subnetwork topology, (b) China aviation subnetwork topology, and (c) China air railway composite network topology.

Figure 1 shows the network topology of the HSR subnetwork, aviation subnetwork, and air railway composite network in mainland China, respectively, where the dot represents the city, and the line indicates the connection between the two cities.

**Figure 2:**
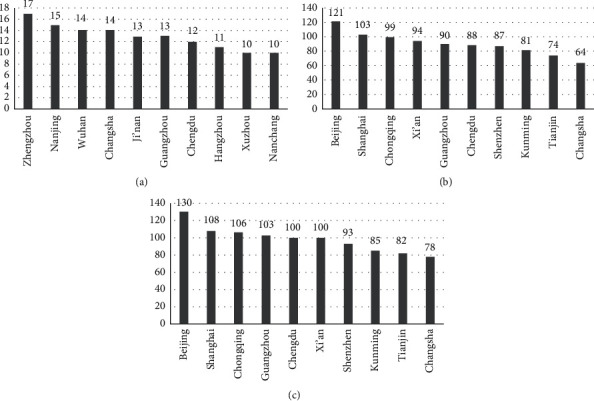
The ranking of the node degrees for the first ten cities for each network topology. (a) Degree numbers and ranking for HSR subnetwork, (b) degree numbers and ranking for aviation subnetwork, and (c) degree numbers and ranking for air railway composite network.

From the network topologies as shown in Figure 1, it is obvious that the HSR subnetwork has apparent community structure characteristics; however, the connection among the communities is relatively loose. While the aviation subnetwork has no obvious community structure, the air railway composite network effectively combines the advantages of the two transport networks, increases the cluster coefficient of the network, and reduces the average path length. Furthermore, the node degree numbers and ranking for the three network topologies mentioned above are shown in Figure 2.

Figures 2(a)–2(c) show ten city nodes with the highest degree of HSR subnetwork, aviation subnetwork, and air railway composite network, respectively. In the HSR subnetwork, Zhengzhou has the largest degree of 17 and 33 city nodes with the degree of 1 accounting for 14.8% of the HSR subnetwork. In the aviation subnetwork, Beijing has the largest degree of 121, and 4 city nodes have the degree of 1 accounting for 2.58% of the aviation subnetwork. The sum of degrees of all city nodes in the HSR subnetwork is 824 and that of all city nodes in the aviation subnetwork is 3,188. According to the 2019 National Statistical Yearbook, the annual passenger volume of the HSR is 2.29 billion and the annual passenger volume of civil aviation is 660 million [6, 36]. Therefore, it can be seen that the transit of civil aviation transportation is more flexible than that of HSR transportation when considering the direct connection among cities. However, HSR transportation has a stronger capability for sending passengers. Moreover, the probability of susceptibility to travel by HSR is slightly lower than that of travel by civil aviation with effective protective measures, but HSR has a greater impact on COVID-19 development than air [37, 38].

**Figure 3:**
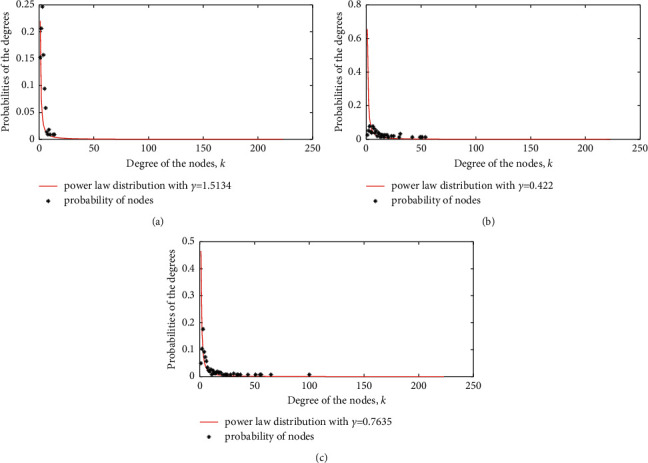
Degree distribution of all three networks. (a) Degree distribution of HSR subnetwork, *γ* = 1.5134, (b) degree distribution of aviation subnetwork, *γ* = 0.422, and (c) degree distribution of air railway composite network, *γ* = 0.7635.

According to equation (1), the power-law fitting, *P*(*k*), of the degree distribution of the nodes over the network roves that the degree distributions of the HSR subnetwork, aviation subnetwork, and air railway composite network follow the different characteristics of the power-law distribution with different *γ*, respectively [39, 40]. The degree distribution analyses are shown in Figure 3.

(1) $$\begin{array}{c} P\left(k\right)∼k^{-\gamma }. \end{array}$$

As shown in Figure 3, it can be seen that the three networks follow the power-law distribution, and the index of the HSR subnetwork degree distribution, *γ*, is 1.5134, the index of the aviation subnetwork degree distribution is 0.422, and the index of the air railway composite network degree distribution is 0.7635. Therefore, they are scale-free and typical heterogeneous networks, which can simulate the virus propagation among cities. At present, although the spread of the COVID-19 virus within mainland China has slowed down, it cannot rule out undetected symptomatic and asymptomatic infections in some cities. The movement of the infected persons over the transportation network may lead other city nodes to be infected. According to reference [41], the prevalence threshold of viruses over heterogeneous networks is defined as

(2) $$\begin{array}{c} \lambda_{c}=\frac{\langle k\rangle }{\langle k^{2}\rangle }, \end{array}$$

where <*k*> is the average degree of the node and *<k*^2^> is the average degree of the second moment, that is,

(3) $$\begin{array}{c} \langle k^{2}\rangle =\frac{1}{N}\overset{N}{\underset{i=1}{\sum }}k_{i}^{2}. \end{array}$$

According to equation (2), it can be seen that as the degree of nodes on the network increases, <*k*^2^>⟶∞, *λ*_*c*_⟶0. When there is virus propagation in the air railway composite network and the average propagation rate on the network *v >λ*_*c*_, the virus will exist on the network for a long time. This means that viruses can easily spread in scale-free networks. We believe that when a node's degree *k*_*i*_ *=* 0, the node is disconnected from other nodes, so the node can be regarded as out of the network. Therefore, the degree of each node over the air railway composite network is greater than 1 and is a positive integer. When the node's degree *k* decreases, the prevalence threshold *λ*_*c*_ over the network will correspondingly increase. The traditional immunization strategies can avoid susceptible units to be infected in advance when the virus spreads; however, the susceptible units described in this study are city nodes and it is not possible to immunize the susceptibility status of city nodes in advance. The method for increasing the prevalence threshold of the composite network mentioned in this study can be adopted to avoid the further spread of the epidemic. The next section will introduce the ARCN-SUTS propagation dynamics model about COVID-19 virus propagating over the network.

## 3. ARCN-SUTS Propagation Dynamics Model

This study considers that the spread of the COVID-19 virus over the air railway composite network leads to the state change of city nodes as the population flow changes and establishes a virus propagation model.

Model assumptions for the state changes of city nodes are placed in this study.

1. This study considers the air railway composite network among city nodes within the scope of mainland China, regardless of the impact of the foreign entrance
2. The asymptomatic infection has a certain latent nature, and the detection rate for this group in cities is lower than that of the symptomatic group
3. The model is suitable for the stable epidemic situation, preventing the second outbreak of the epidemic over the air railway composite network and a more serious impact
4. assumed that no matter what scale of infection or asymptomatic infection occurs in a certain city node, it will immediately lead to state changes of city nodes and employ corresponding strategies

**Figure 4:**
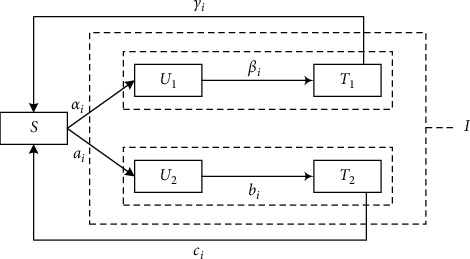
Schematic diagram of ARCN-SUTS propagation model.

Population mobility is common among cities, especially among large cities. Generally, city nodes over a composite network are classified into three states including the state vulnerable to infection (susceptible, *S*), the state existing untested infected persons (untested, *U*), and a state existing tested infected persons (tested, *T*). Due to the population movement in a city and the spread of the COVID-19 virus from person to person, the status of the city nodes will change. As shown in the schematic diagram of the COVID-19 virus propagation model among cities in Figure 4, if there is no infected person in a city, it means that the city node is safely located in the susceptible state, *S*. If an untested infected person appears in a city, the city is located in an untested state, *U*, at this time. After using certain testing methods, the infected person is found in a city; then, the city is located in a tested state, *T*. Even if the infected city recovers, it may be infected again within a certain period of time; therefore, this city is still located in the state, *S*. In this case, this study proposes a “susceptible-untested-tested-susceptible” (ARCN-SUTS) model applied to the air railway composite network, which can better describe the spread of the virus among cities. The schematic diagram of the proposed propagation model is shown in Figure 4.

It is known that COVID-19 infection can be divided into two types: symptomatic infection and asymptomatic infection. The susceptible city node *S* can change into the state *U*_1_ where the symptomatic untested infection persons exist. At this time, the nodes in state *U*_1_ begin to infect other nodes over the network with propagation rate *α*_*i*_. The city constantly checks the infected person through some testing methods. If the city node locates in the state *U*_1_, it can change from the state *U*_1_ to the state *T*_1_ with the detection rate, *β*_*i*_, where *T*_1_ is the state with the tested symptomatic infection. Once the infected persons are found in the city, they will be immediately treated until all the infected persons are cured. Then, the city returns to the susceptible state *S* from state *T*_1_ with the rate *γ*_*i*_. Similarly, the susceptible city node *S* can also change into the state *U*_2_ where asymptomatic untested infection persons exist. The nodes in state *U*_2_ begin to infect other nodes over the network with the propagation rate *a*_*i*_. If the city node is located in the state *U*_2_, it can change from the state *U*_2_ to the state *T*_2_ with the testing rate, *b*_*i*_, where the state *T*_2_ is the tested asymptomatic infection. Then, the city returns to the susceptible state *S* from state *T*_2_ with rate *c*_*i*_. *U*_1_ and *U*_2_ are hidden states, and *T*_1_ and *T*_2_ are dominant states.

The propagation dynamic model of ARCN-SUTS is shown as follows:

(4) $$\begin{array}{c} \left\{\begin{array}{c} \frac{\text{d}s\left(t\right)}{\text{d}t}=\gamma_{i}T_{1}\left(t-\tau_{i}\right)+c_{i}T_{2}\left(t-\tau_{i}\right)-\alpha_{i}S\left(t\right)U_{1}\left(t\right)-a_{i}S\left(t\right)U_{2}\left(t\right)\\ \frac{\text{d}U_{1}\left(t\right)}{\text{d}t}=\alpha_{i}S\left(t\right)U_{1}\left(t\right)-\beta_{i}U_{1}\left(t\right)\\ \frac{\text{d}T_{1}\left(t\right)}{\text{d}t}=\beta_{i}U_{1}\left(t\right)-\gamma_{i}T_{1}\left(t-\tau_{i}\right)\\ \frac{\text{d}U_{2}\left(t\right)}{\text{d}t}=a_{1}S\left(t\right)U_{2}\left(t\right)-b_{i}U_{2}\left(t\right)\\ \frac{\text{d}T_{2}\left(t\right)}{\text{d}t}=b_{i}U_{2}\left(t\right)-c_{i}T_{2}\left(t-\tau_{i}\right) \end{array}\right., \end{array}$$

where

 
*α*_*i*_ is the symptomatic infection rate of each city, 0 < *α*_*i*_ < 1, *iϵ* {1, 2,…, *num*}, and *num* is the number of city nodes.
 
*β*_*i*_ is the symptomatic infection testing rate in each city associated with the virus testing level of city *i*, 0 < *β*_*i*_ < 1, *i* *ϵ* {1, 2,…,*num*}.
 
*γ*_*i*_ represents the recovery rate of a city for the city changing from testing symptomatic infection state into a susceptible state associated with the medical treatment level of city *i*, 0 < *γ*_*i*_ < 1, *i* *ϵ* {1, 2,…,*num*}.
 
*a*_*i*_ refers to the asymptomatic infection rate of each city, 0 < *a*_*i*_ < 1, *i* *ϵ* {1, 2,…,*num*}.
 
*b*_*i*_ is the testing rate for asymptomatic infection in each city associated with the testing level of city *i*, 0 < *b*_*i*_ < 1, *i* *ϵ* {1, 2,…,*num*}.
 
*c*_*i*_ is the recovery rate for the city changing from the testing asymptomatic infection state to a susceptible state associated with the medical treatment level of city *i*, 0 < *c*_*i*_ < 1, *i* *ϵ* {1, 2,…,*num*}.
 
*τ*_*i*_ is the time delay. It takes some time for city *i* to recover from infection states, *T*_1_ or *T*_2_. *τ*_*i*_ refers to the shortest time for all infected persons in city *i* to be cured.
 
*α*_*i*_ and *a*_*i*_ represent the infectious rate for symptomatic infection and asymptomatic infection, respectively. These parameters also indicate city state change due to the population flow over the transport network. The mobility of viruses among city nodes can be regarded as the mobility of viruses carried by all travelers. The greater the external passenger flow, the higher the external propagation capacity of the node city.

This section uses the SIR virus propagation model among people and the real data for COVID-19 to obtain the parameters for SIR and then considers other factors to obtain the parameters more accurately mentioned above. This section will describe the procedures to obtain the corresponding parameters in detail. It is known that there are 5 types of states among people, namely, susceptible, explored, asymptomatic infected, symptomatic infected, and recovered. Among them, explored, asymptomatic, and symptomatic infected persons are classified as infection status.

Model assumptions for the propagation model among people are placed as follows:

1. In reality, patients in the incubation stage are classified as infections
2. The model only considers the patients who are tested, treated, and cured in the same city and does not consider the patients transited from other cities
3. The whole curing period for a city starts from the first patient's treated to the last patient cured
4. The model does not consider reinfection cases

**Figure 5:**
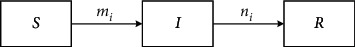
Schematic diagram of COVID-19 virus SIR propagation model among people.

Figure 5 depicts the SIR propagation model of the COVID-19 virus in people.

 
*S*, *I*, and *R* are defined as the susceptible, the infectious, and the recovered.
 
*m*_*i*_ is the infection rate of the virus in city *i*, 0 < *m*_*i*_ < 1, *iϵ*{1, 2,…,*num*}. *N*_*i*_ is the cure rate of infected persons in city *i*, 0 < *n*_*i*_ < 1, *iϵ*{1, 2,…,*num*}.

Therefore, the dynamic model for COVID-19 virus SIR propagation among people is established as follows:

(5) $$\begin{array}{c} \left\{\begin{array}{c} \frac{\text{d}S\left(t\right)}{\text{d}t}=-m_{i}S\left(t\right)I\left(t\right)\\ \frac{\text{d}I\left(t\right)}{\text{d}t}=m_{i}S\left(t\right)-n_{i}I\left(t\right)\\ \frac{\text{d}R\left(t\right)}{\text{d}t}=n_{i}I\left(t\right) \end{array}\right.. \end{array}$$

**Figure 6:**
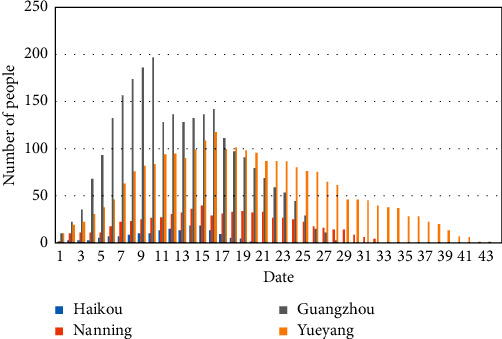
The statistical analysis of the number of infected persons for different cities.

According to the daily number of diagnosed and recovered people in each city, the number changes of the infected persons in all cities are obtained.  Figure 6 shows the analysis of the changes in the number of infected persons in some cities.

As shown in Figure 6, it takes about 15 days from the first tested infection to the peak number for each city, and the number gradually decreases with the increase in isolation measures and continuous treatments. The corresponding fitted parameters *m*_*i*_ and *n*_*i*_ by using the least-squares method are shown in Table 1.

According to references [42–44], the degree of the city nodes over the composite network, *k*, and total passenger flows, *f*, can determine the relationships among *α*_*i*_ and *m*_*i*_, and *γ*_*i*_ and *n*_*i*_. The relationships are described in equation (6), where *f*_*i*_ is the flow of passengers passing through the city *i*. The corresponding *α*_*i*_ and *γ*_*i*_ values for the selected cities are shown in Table 2.

(6) $$\begin{array}{c} \left\{\begin{array}{c} \alpha_{i}=m\times \frac{k_{i}\times f}{k\times f_{i}}\\ \gamma =n_{i}\times \frac{k_{i}\times f}{k\times f_{i}} \end{array}.\right. \end{array}$$

Next, as reported, the relationships between *a*_*i*_, *α*_*i*_, and *b*_*i*_, *β*_*i*_ are described as

(7) $$\begin{array}{c} a_{i}=\left(\frac{1}{3}\right)\alpha_{i},\\ b_{i}=\left(\frac{1}{3}\right)\beta_{i}. \end{array}$$

The testing rate *β*_*i*_ of the city *i* is related to city size, which is characterized by city GDP (data coming from website [6]). *β*_*i*_ is calculated according to equation (8).

(8) $$\begin{array}{c} \beta_{i}=\frac{D_{n}}{\text{pop}}\times \frac{{\text{GDP}}_{i}}{\bar{\text{GDP}}}, \end{array}$$

where *D*_*n*_ is the number of the current daily testing in China, *pop* is the current population of China, *GDP*_*i*_ is GDP of city *i*, and $\bar{\text{GDP}}$ is the average GDP of all cities in China. Therefore, *β*_*i*_ will float according to the city size. As reported, the cure rate of asymptomatic infection is slightly higher than that of symptomatic infection; therefore, in this study, *γ*_*i*_ = (1 − *r*)*c*_*i*_, *r* is a random number, and 0 < *r* < 0.1.

## 4. Broken-Edge Decision-Making Strategy

**Figure 7:**
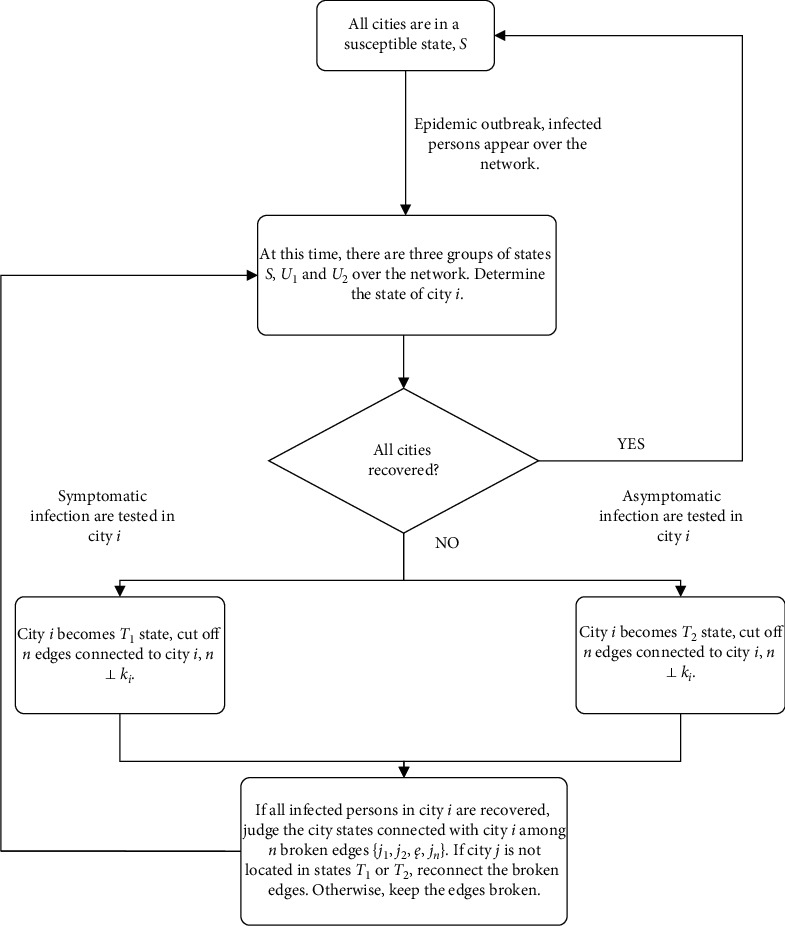
Schematic diagram of broken-edge decision-making strategy.

Based on the analysis of the degree of the air railway composite network node in Section 2, it is known that the greater the degree *k* is, the smaller the epidemic threshold *λ*_*c*_ is. In order to avoid the second outbreak of the epidemic, this study puts forward a broken-edge decision-making strategy and analyzes the controllability of the epidemic spread over the air railway composite network. Among them, the schematic diagram of the broken-edge decision-making strategy is shown in Figure 7.

As illustrated in Figure 7, during the epidemic duration, when a symptomatic or asymptomatic infection is tested in the city *i* over the air railway composite network, the state of city *i* changes into state *T*_1_ or state *T*_2_; then, cut off the edges are connected with city *i* according to the strategy. If all the infected persons in city *i* recover, simultaneously, the broken-edge cities *j* are not located in states *T*_1_ or *T*_2_, then the state of city *i* returns to state *S* and reconnects the broken edges among cities *i* and *j*. The broken-edge strategy over the air railway composite network can ensure network controllability; however, it is inevitable to cause economic losses for aviation and railway companies. Therefore, it is necessary to minimize economic losses on the premise of virus propagation controlled. The objective function and constraints are shown as follows:

(9) $$\begin{array}{c} \min P_{\text{Loss}}=\sum_{i=1}\sum_{j=1}e_{\text{ij}}x_{\text{ij}}w_{\text{ij}},\\ s.t.\left\{\begin{array}{c} e_{\text{ij}}\geq 0\\ w_{\text{ij}}\geq 0\\ x_{\text{ij}}=x_{\text{ij}}\\ x_{\text{ij}}=0\text{ or }1 \end{array},\right. \end{array}$$

where *e*_*ij*_ is the unit freight rate from city *i* to city *j*, *w*_ij_ is the total transport volume from city *i* to city *j*, and *x*_*ij*_ is a binary variable. If the connected edge between city *i* and city *j* is broken, *x*_*ij*_ = 1; otherwise, it is 0.

The ergodic optimization algorithm of the broken-edge decision-making strategy is adopted to minimize the economic loss over the air railway composite network, and the strategy algorithm is illustrated in Table 3.

Based on the broken-edge decision-making strategy proposed in this study, it can effectively cut off the connection between the outbreak city *i* and some susceptible cities *j*. In Section 5, the simulations are conducted to analyze the effectiveness of the proposed strategy.

## 5. Simulation Analyses

The air railway composite network with 261 city nodes proposed in Section 2 is considered, and cities with different states are randomly set. The initial numbers of cities for different states are shown in Table 4.

The state transition of each city for the next moment is determined by using the ARCN-SUTS propagation dynamics model (3). The transmission rate and recovered rate in the model (3) are obtained by using fitting model (4) with actual data. Then, the experiment carries out the broken-edge decision-making strategy mentioned in Section 4, and Monte Carlo runs are conducted 32,769 times.

**Figure 8:**
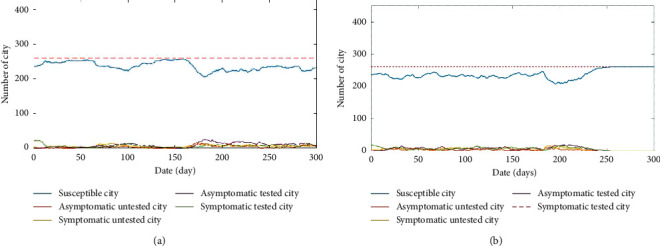
Comparisons of node states with or without the broken-edge decision-making strategy over a composite network. (a) The numbers of nodes for different state changes without using the broken-edge decision-making strategy. (b) The numbers of nodes for different state changes with using the broken-edge decision-making strategy.

Under the same initial conditions, Figure 8 demonstrates the number change of cities in different states over the air railway composite network with and without the broken-edge decision-making strategy, respectively. As seen from Figures 8(a) and 8(b), without adopting the broken-edge decision-making strategy, the epidemics will continue to spread across the entire network and cannot be completely controlled. When using the broken-edge decision-making strategy, all cities can be moved to the state *S* after approximately 256 days. It further demonstrates that the broken-edge decision-making immunization strategy can effectively shorten the recovery time of all cities to susceptible status and thus control the spread of the epidemic by the simulation comparisons.

**Figure 9:**
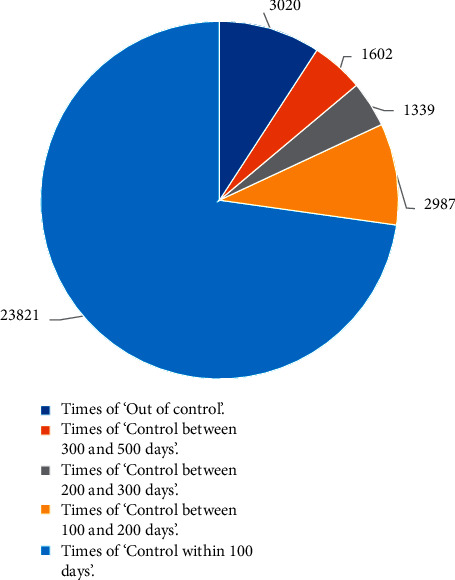
Effect analysis of epidemic control for the simulation.

As shown in Figure 9, among the 32,769 simulations, the number of epidemic-controlled cases accounts for 90.78% in total, where the number of epidemics controlled within 100 days cases accounts for 72.69%, and the number of epidemics uncontrolled cases accounts for 9.22%. Therefore, this model effectively controls the COVID-19 epidemics. The ergodic algorithm was further used to calculate the shortest time for all city nodes recovered to the susceptible state *S* in the air railway composite network with the broken-edge decision-making strategy.

**Figure 10:**
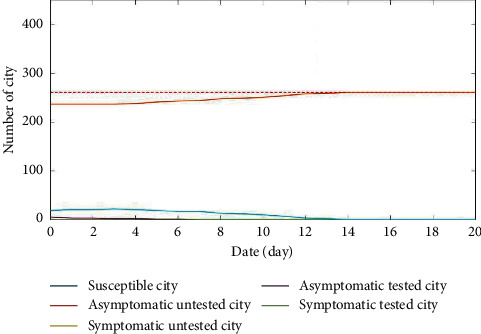
The number changes of cities in different states under the control strategy with the shortest epidemic duration.

Under the control strategy with the shortest epidemic duration, Figure 10 demonstrates the number changes of cities in various states. It is obvious that the shortest time for all city nodes recovered to state *S* is 14 days. The broken-edge strategies for some city nodes are listed in Table 5.

According to the data analysis in Table 5, in the initial stage of the outbreak, the appropriate number of edges was cut off on the nodes of cities in states *T*_1_ and *T*_2_ to quickly curb the outbreak. When the virus epidemic becomes stable, the number of broken edges will be gradually reduced, and finally the air railway composite network will be stable and safe. The average times to control the epidemics for different cases are obtained through the broken-edge decision-making strategy as shown in Table 6. Figure 9 shows that the broken-edge decision-making strategy has a 90.78% probability of controlling the duration of the epidemic within 100 days, and the average duration of the epidemic is 42.0358 days as shown in Table 6.

The shortest epidemic duration with this strategy is 14 days as shown in Figure 10. The minimum time is far less than the average duration for controlling epidemics within 0∼100 days as shown in Table 6, so it can be concluded that the broken-edge decision-making strategy control effect is better. Furthermore, on the premise of controlling the average propagation rate on the network, *v*, that is, *v* ＜ *λc*, the objective and constraint functions (8) will be taken into account, and the total economic losses are minimized for aviation and railway companies by using the broken-edge decision-making strategy.

**Figure 11:**
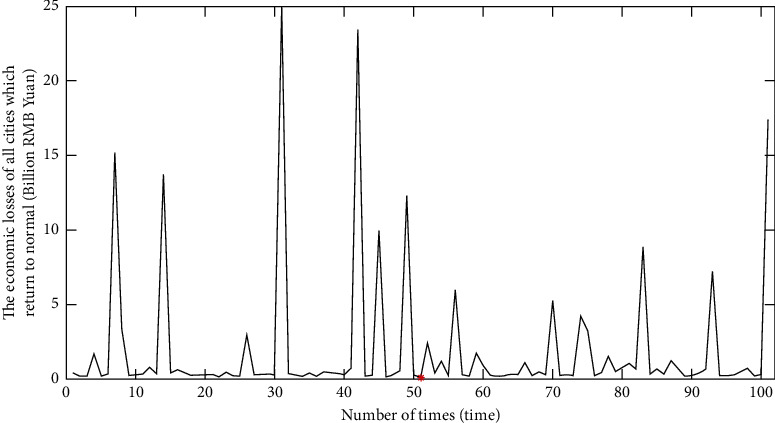
Partial areas of minimum economic losses with the broken-edge decision-making strategy.

**Figure 12:**
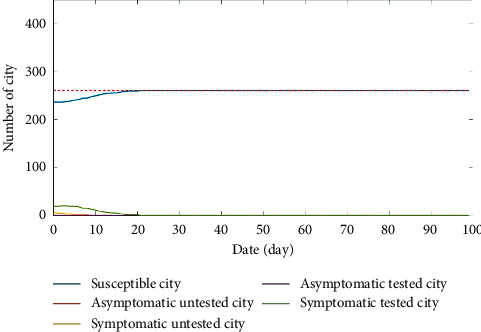
The number changes of cities in each state ensure the minimum economic loss.

Partial areas of minimum economic losses for aviation and railway companies with broken-edge decision-making strategies are shown in Figure 11. The red dot represents the least economic losses over the whole time frame of the epidemic spread simulation, and it is the global optimization point.  Figure 12 demonstrates the variation of the city node number in each state with minimum economic loss broken-edge decision-making strategy. As illustrated in Figure 11, the total minimum loss is 944.9 million RMB yuan. Correspondingly, it takes 21 days to completely control the epidemics. From another perspective, the total loss is 2.55 billion RMB yuan with the shortest epidemic duration, and it takes 14 days to completely control the epidemics.

Table 7 lists the average economic losses for aviation and railway companies during different time frames. According to Table 7, the minimum economic loss happens within 100 days under the epidemics controlled, which is far less than that of other time frames. As analyzed in Tables 5 and 7, the average losses within the short time frame are less than those within the long time frame. Therefore, it is recommended to cut off the multiple edges in the initial stage within a reasonable range. As the epidemic becomes stable, it can reduce the number of broken edges in order to control the spread of the COVID-19 virus in the early stage and less economic losses for airlines and railway companies.

## 6. Conclusions

To control the spread of the epidemics over the air railway composite network and minimize the economic loss of airlines and railway companies, this study proposes an efficient decision-making strategy after the outbreak of COVID-19. The main works of this study are summarized as follows. First, this study considers an air railway composite network composed of prefecture-level cities and above in mainland China and analyzes the degree and degree distribution of the networks, which verify the scale-free characteristics of the networks. The traffic flow among city nodes can be more accurately simulated over the air railway composite network. Second, this study establishes the ARCN-SUTS model based on the propagation characteristics of the COVID-19 virus among city nodes over the air railway composite network, which more objectively and rationally describes the variations of city states. Third, the broken-edge decision-making strategy is proposed to control the spread of the virus in the composite network with less time and minimize economic losses. It effectively solves the problem of dynamic emergency decision-making for the spread of the COVID-19 epidemic based on traffic networks. The effect of the strategy is introduced from the economic benefits and the time benefit assessment for controlling COVID-19. Furthermore, the corresponding simulation results show the effectiveness of the broken-edge decision-making strategy for controlling the spread of COVID-19 among cities over the air railway composite network. However, this study has not conducted a comprehensive study on the control methods of epidemic transmission between urban nodes through transportation networks. Therefore, it is not yet possible to effectively make a comparative analysis among multiple control methods. The focus of future research will enhance the development of control strategies in this area.

## Figures and Tables

**Table 1:** Infection rate and recovery rate for some cities.

*Probability*	*City*_*i*_						
	Neijiang	Leshan	Nanchong	Meishan	Yibin	…	City_k_
*m*_*i*_	0.8926405	0.6321406	0.5060117	0.1272747	0.3610853	…	…
*n*_*i*_	0.0606787	0.003846	0.0065079	0.0020471	0.0034049	…	…

**Table 2:** Infection rate and recovery rate for some cities.

*Probability*	*City*_*i*_						
	Neijiang	Leshan	Nanchong	Meishan	Yibin	…	City_k_
*α*_*i*_	0.6498423	0.525941	0.6679355	0.3347325	0.3213659	…	…
*γ*_*i*_	0.0441741	0.0031999	0.0085904	0.0053838	0.0030304	…	…

**Table 3:** Strategy algorithm.

Step 1	Randomly generate cities in different states, set the maximum time limit for controlling epidemics, *t*_max_, and set the number of iterations, *n.*
Step 2	Filter out nodes located in states *T*_1_ and *T*_2_ and find out all possible broken edges. Randomly generate recovered periods *τ*_*i*_.
Step 3	Randomly cut off partially broken edges and record them.
Step 4	Calculate the current economic losses.
Step 5	Determine each node state for the next time according to the ARCN-SUTS model. Cities located in state *T*_1_ or state *T*_2_ will return to state *S* at the next moment. If the state for the broken-edge city is not state *T*_1_ or state *T*_2_, reconnect the broken edge; otherwise, keep broken.
Step 6	If the number of nodes in state *S* is equal to the total number of nodes, go to step 7. If the number of nodes in state *S* is not equal to the total number of nodes, and the date has not reached the maximum time limit *t*_max_ for controlling the epidemics, go to step 2. If the date reaches the maximum time limit *t*_max_ to control the epidemic, go to step 8.
Step 7	Record the total economic losses and date.
Step 8	Keep the initial city state; if the set iteration number, *n*, is not reached, go to step 2; otherwise, end.

**Table 4:** Initial number of cities for different states.

Different states of the city	Susceptible city	Asymptomatic untested city	Symptomatic untested city	Asymptomatic tested city	Symptomatic tested city
Number of cities	237	0	5	0	19

**Table 5:** Number of broken edges and partially broken edges of city nodes under the broken-edge strategy with the shortest epidemic duration.

(a) Number of broken edges per day							

Date	1	2	3	4	5	6	7
Number of edges	85	11	0	3	0	3	0
Date	8	9	10	11	12	13	14
Number of edges	11	0	0	0	0	0	0

(b) Broken-edge decision-making strategy for some city nodes							
Date	1			2			
Broken lines	Xinzhou-Nanjing	…	Tongren-Kunming	Liaoyang-Panjin	…	Chizhou-Anqing	
Date	…			8			
Broken lines	…			Guangzhou-Nanyang	…	Tianjin-Nanyang	

**Table 6:** Average duration for controlling epidemics in different time frames.

The time frame for controlling epidemics (days)	0～100	100～200	200～300	300～500
Average duration of the epidemic (days)	42.0358	140.6294	246.1509	391.4145

**Table 7:** Average economic losses in different time frames.

The time frame for controlling epidemics (days)	0～100	100～200	200～300	300～500
Average loss (billion yuan)	4.91	39.02	94.11	168.98

## Data Availability

The data used to support the findings of this study are included within the article.

## References

[1] White paper-fighting COVID-19: China in action (2020). https://covid-19.chinadaily.com.cn/a/202006/08/WS5edd8bd6a3108348172515ec.html.

[2] Coronavirus disease 2019 (COVID-19) situation report - 73 (2020). https://www.who.int/docs/default-source/coronaviruse/situation-reports/20200402-sitrep-73-covid-19.pdf?sfvrsn=5ae25bc7_6.

[3] Wei F., Wang J., Xu X. (2020). Tendency prediction of COVID-19 worldwide. *Disease Surveillance*.

[4] Zhao X., Li X., Nie C. (2020). Backtracking Transmission of COVID-19 in China Based on Big Data Source, and Effect of Strict Pandemic Control Policy. *Bulletin of Chinese Academy of Sciences*.

[5] Zhang L., Li D., Ren J. (2020). Analysis of COVID-19 by Discrete Multi-stage Dynamics System with Time Delay. *Journal of Wuhan University (Information Science Edition)*.

[6] National Bureau of Statistics http://www.stats.gov.cn/.

[7] Xu H., Zhang Q. (2020). Epidemic Dynamics on Complex Networks: A Review. *Information Science*.

[8] Li Z., Gou C. (2020). Stability analysis of a class of SIR endemic model with diffusion. *Journal of Fuzhou University (NATURAL SCIENCE EDITION)*.

[9] Bjørnstad ON, Shea K, Krzywinski M, Altman N (2020). The SEIRS model for infectious disease dynamics. *Nature methods*.

[10] Jin M., Lin Y. (2021). Classification of asymptotic behavior in a stochastic SEIR epidemic model. *Applied Mathematics Letters*.

[11] Chen G., Xiao M., Wan Y., Wang X. (2021). Propagation dynamics of fractional order delay epidemic model. *Control Theory & Applications*.

[12] Chen S., Small M., Tao Y. (2016). Transmission dynamics of an SIS model with age structure on heterogeneous networks. *Bulletin of Mathematical Biology*.

[13] Ding Q., Li W., Hu X., Zheng Z., Tang S. (2020). The SIS diffusion process in complex networks with independent spreaders. *Physica A: Statistical Mechanics and its Applications*.

[14] Pastor-Satorras R., Vespignani A. (2001). Epidemic Spreading in Scale-Free Networks. *American Physical Society*.

[15] Liu Y.-Y., Slotine J.-J., Barabási A.-L. (2011). Controllability of Complex Networks. *Nature*.

[16] Hou L., Lao S., Xiao Y., Bai L. (2015). Recent progress in controllability of complex network. *Acta Physica Sinica*.

[17] Mo H. (2020). An Emergency Decision-Making Method for Probabilistic Linguistic Term Sets Extended by D Number Theory. *Symmetry*.

[18] Fu M., Wang L., Zhu J., Zheng B. (2021). Emergency Optimization Decision-Making with Incomplete Probabilistic Information under the Background of COVID-19. *Complexity*.

[19] Li M.-Y., Cao P.-P. (2019). Extended TODIM method for multi-attribute risk decision making problems in emergency response. *Computers & Industrial Engineering*.

[20] Amorim M., Antunes F., Ferreira S., Couto A. (2019). An integrated approach for strategic and tactical decisions for the emergency medical service: Exploring optimization and metamodel-based simulation for vehicle location. *Computers & Industrial Engineering*.

[21] Wang Y., Liang Y., Sun H., Yang Y. (2021). Decision-Making for Fire Emergency of Urban Rail Transit Based on Prospect Theory. *Mathematical Problems in Engineering*.

[22] Wan S.-P., Huang Cheng W.-B., Dong J.-Y. (2021). Interactive multi-criteria group decision-making with probabilistic linguistic information for emergency assistance of COVID-19. *Applied Soft Computing*.

[23] Wan S.-p., Chen Z.-h., Dong J.-y. (2021). Bi-objective trapezoidal fuzzy mixed integer linear program-based distribution center location decision for large-scale emergencies. *Applied Soft Computing*.

[24] Givoni M., Banister D. (2006). Airline and railway integration. *Transport Policy*.

[25] Xu F., Zhu J., Miao J., Ding R. (2018). Simulation of Two Stages-Variant Growth Evolution Model of High-speed Railway and Civil Aviation Compound Network. *Journal of System Simulation*.

[26] Xia W., Zhang A. (2016). High-speed rail and air transport competition and cooperation: A vertical differentiation approach. *Transportation Research Part B: Methodological*.

[27] Yang X., Wang Z., Yang Y., Qin Y. (2016). Constructing Hub and Spoke Airway-railway Composite Traffic Network in China. *China Transportation Review*.

[28] Ye Y., Li W., Zhang J. (2019). Complex Characteristics and Propagation Dynamics of High Speed Railway Network. *Journal of Tongji University (NATURAL SCIENCE EDITION)*.

[29] Tang Z., Huang S., Han S. (2021). Recent Progress about Flight Delay under Complex Network. *Complexity*.

[30] Wu W., Wu C., Feng T., Zhang H., Qiu S. (2018). Comparative Analysis on Propagation Effects of Flight Delays: A Case Study of China Airlines. *Journal of Advanced Transportation*.

[31] Zhong P., Yin H. (2020). System dynamics simulation on spread of COVID-19 by traffic and transportation. *Journal of Traffic and Transportation Engineering*.

[32] Elhiwi M. (2021). Stochastic Model for the Spread of the COVID-19 Virus. *Applied Mathematics*.

[33] Li J., Xiang T., He L. (2021). Modeling epidemic spread in transportation networks: A review. *Journal of Traffic and Transportation Engineering (English Edition)*.

[34] China Railway Customer Service Center https://www.12306.cn/index/.

[35] Umetrip Information Search http://www.umetrip.com/mskyweb/main/index.html.

[36] 2019 China Civil Aviation Passenger Traffic Civil Aviation Resources (2020). http://news.carnoc.com/list18065.html.

[37] Zhang Y., Wang X., Bi Q. (2020). Travel-Infected Susceptibility Based on Transmission Mechanism of COVID-19. *Transport Research*.

[38] Zhu P., Guo Y. (2021). The Role of High-speed Rail and Air Travel in the Spread of COVID-19 in China. *Travel medicine and infectious disease*.

[39] Wang X. (2020). Controversial Issues in Researches on Scale-free Networks: An Overview with a Network Perspective. *Journal of University of Electronic Science and Technology of China*.

[40] Xu F., Zhu J., Yang W. (2013). Construction of High-speed Railway and Airline Compound Network and the Analysis of Its Network Topology Characteristics. *Complex System and Complexity Science*.

[41] Pastor-Satorras R., Vespignani A. (2002). Immunization of complex networks. *Physical Review E: Statistical, Nonlinear & Soft Matter Physics*.

[42] Zhang Y., Zhang A., Wang J. (2020). Exploring the roles of high-speed train, air and coach services in the spread of COVID-19 in China. *Transport Policy*.

[43] Chinazzi M., Davis J. T., Ajelli M. (2020). The effect of travel restrictions on the spread of the 2019 novel coronavirus (COVID-19) outbreak. *Science*.

[44] Fang H., Wang L., Yang Y. (2020). Human mobility restrictions and the spread of the novel coronavirus (2019-nCoV) in China. *Journal of Public Economics*.

